# DNA Methylation Basis in the Effect of White Matter Integrity Deficits on Cognitive Impairments and Psychopathological Symptoms in Drug-Naive First-Episode Schizophrenia

**DOI:** 10.3389/fpsyt.2021.777407

**Published:** 2021-12-13

**Authors:** Xiaofen Zong, Qinran Zhang, Changchun He, Xinyue Huang, Jiangbo Zhang, Gaohua Wang, Luxian Lv, Deen Sang, Xiufen Zou, Huafu Chen, Junjie Zheng, Maolin Hu

**Affiliations:** ^1^Department of Psychiatry, Renmin Hospital of Wuhan University, Wuhan, China; ^2^Department of Psychiatry, The Second Xiangya Hospital, Central South University, Changsha, China; ^3^School of Mathematics and Statistics, Wuhan University, Wuhan, China; ^4^Hubei Key Laboratory of Computational Science, Wuhan University, Wuhan, China; ^5^High-Field Magnetic Resonance Brain Imaging Key Laboratory of Sichuan Province, School of Life Science and Technology, University of Electronic Science and Technology of China, Chengdu, China; ^6^Department of Psychiatry, Henan Mental Hospital, The Second Affiliated Hospital of Xinxiang Medical University, Xinxiang, China; ^7^Early Intervention Unit, Department of Psychiatry, Affiliated Nanjing Brain Hospital, Nanjing Medical University, Nanjing, China; ^8^Functional Brain Imaging Institute, Nanjing Medical University, Nanjing, China

**Keywords:** schizophrenia, diffusion tensor imaging, DNA methylation, cognitive function, Allen Human Brain Atlas

## Abstract

**Background:** Mounting evidence from diffusion tensor imaging (DTI) and epigenetic studies, respectively, confirmed the abnormal alterations of brain white matter integrity and DNA methylation (DNAm) in schizophrenia. However, few studies have been carried out in the same sample to simultaneously explore the WM pathology relating to clinical behaviors, as well as the DNA methylation basis underlying the WM deficits.

**Methods:** We performed DTI scans in 42 treatment-naïve first-episode schizophrenia patients and 38 healthy controls. Voxel-based method of fractional anisotropy (FA) derived from DTI was used to assess WM integrity. Participants' peripheral blood genomic DNAm status, quantified by using Infinium® Human Methylation 450K BeadChip, was examined in parallel with DTI scanning. Participants completed Digit Span test and Trail Making test, as well as Positive and Negative Syndrome Scale measurement. We acquired genes that are differentially expressed in the brain regions with abnormal FA values according to the Allen anatomically comprehensive atlas, obtained DNAm levels of the corresponding genes, and then performed Z-test to compare the differential epigenetic-imaging associations (DEIAs) between the two groups.

**Results:** Significant decreases of FA values in the patient group were in the right middle temporal lobe WM, right cuneus WM, right anterior cingulate WM, and right inferior parietal lobe WM, while the significant increases were in the bilateral middle cingulate WM (*P*s < 0.01, GRF correction). Abnormal FA values were correlated with patients' clinical symptoms and cognitive impairments. In the DEIAs, patients showed abnormal couple patterns between altered FA and DNAm components, for which the enriched biological processes and pathways could be largely grouped into three biological procedures: the neurocognition, immune, and nervous system.

**Conclusion:** Schizophrenia may not cause widespread neuropathological changes, but subtle alterations affecting local cingulum WM, which may play a critical role in positive symptoms and cognitive impairments. This imaging-epigenetics study revealed for the first time that DNAm of genes enriched in neuronal, immunologic, and cognitive processes may serve as the basis in the effect of WM deficits on clinical behaviors in schizophrenia.

## Introduction

Schizophrenia is a severe mental disorder defined by a disruption in emotion and cognition along with positive and negative symptoms (1). Mounting evidence, such as neuropathological connectivity alterations from magnetic resonance imaging (MRI) studies, supports the neurodevelopmental model of schizophrenia, in which prenatal viral infection is proposed to result in activation of DNA methylation process, maternal immune response, as well as altered expression of genes involved in multiple signaling systems, and subsequent genesis of schizophrenia (2, 3). White matter (WM) connectivity abnormalities have been suggested to be the potential biomarkers for the diagnosis of schizophrenia (4, 5). Diffusion tensor imaging (DTI) can *in vivo* explore the WM structure and pathology (6). Fractional anisotropy (FA), derived from DTI, has been proposed to reflect microstructural integrity of WM, including the density of axonal fibers, the extent of myelination, and axonal architecture (7, 8). Accumulating DTI studies of individuals with schizophrenia have described abnormalities in the WM microstructure as inferred by decreased or increased FA that are spatially extensive but may demonstrate regional variations in severity with prefrontal, temporal, parietal, and cingulate WM being more affected (9). Moreover, abnormal alterations of FA showed significant associations with the severity of patients' positive and negative symptoms (5), as well as impaired cognitive function such as working memory, executive function, verbal fluency, speed of processing, motor speed, and attention (10, 11). The robust evidence reveals that abnormal alterations of WM integrity may be potential biomarkers for the diagnosis of schizophrenia (12), and serve as a neuropathological foundation for clinical symptoms and neurocognitive function impairments in this disease (13).

Epigenetic modifications, mediating between genes and environment, have been considered to be involved in the pathogenesis of schizophrenia (14). DNAm is a well-characterized and stable epigenetic mechanism. Frequently reported DNAm alterations associated with schizophrenia pathology are mainly enriched in biological processes or pathways such as neural signaling, metabolic, cognitive, and immune systems (15–18). It is worth noting that neuroimaging-epigenetic studies can provide crucial and complementary information about epigenetics and intermediate phenotypes for schizophrenia, although most existing studies focused on either of one modal dataset analysis. A combined method simultaneously exploring how DNAm variances lead to neuroimaging changes, as well as how abnormal brain imaging phenotypes relate to clinical symptoms and cognitive impairments in the same schizophrenia sample, would prove more informative in understanding such a complex illness.

A study combining genomic DNAm and regional brain volumes derived from MRI showed that the alterations in the methylation of the genes enriched in cerebellum development and neuronal growth may contribute to the decrease of cerebellar volume in schizophrenia patients (19). Recent epigenetic imaging studies revealed the associations between WM microstructural deficits and DNAm modification in major depressive disorder (20, 21), implying that impairments of WM integrity in neuropsychic disease may be epigenetically regulated, although this hypothesis remains untested in schizophrenia. A meta-analysis of epigenomic association studies of cognitive function proposed that the DNAm modification may provide insight into the link between perturbed alterations of FA and cognitive function (22). However, the associations among DNAm, WM microstructure abnormalities in schizophrenia, as well as cognitive impairments and psychopathological symptoms, have not yet been explored.

To exclude the influence of confounders such as disease progression and long-term antipsychotic medication, this study would focus on drug-naïve first-episode schizophrenia patients. Voxel-based analysis (VBA), a voxel-by-voxel and whole-brain analytic approach, was conducted to compare FA between patients and healthy controls. Although VBA will allow us to detect WM alterations anywhere they might occur, given the previous evidence (5, 9, 10), our specific hypothesis is that patients with first-episode schizophrenia would show abnormal FA located in prefrontal, temporal, parietal, and cingulate WM, and the abnormalities would correlate with patients' clinical behaviors. Support vector machine (SVM) analysis, an optimized classification method, was used to explore the accuracy of abnormal FA values in distinguishing patients from healthy volunteers. Given evidence supporting the associations between the peripheral blood DNAm status and schizophrenia pathogenesis (18), as well as a “mirror” pattern that the DNAm state in the blood cells is related to that of the corresponding disease-causing regions in the brain (23), our current study would assess the genome-wide DNAm status derived from peripheral blood cells. Then, we acquired the genes lists that are differentially expressed in the brain regions with abnormal FA values according to the Allen anatomically comprehensive atlas (24), and then calculated the possible associations between the abnormal FA and DNAm levels corresponding to the genes lists. Based on the neurodevelopmental hypothesis of schizophrenia (2), we hypothesized that the impairments of WM integrity in patients would be regulated by methylation modifications of genes enriched in brain development and/or immune process pathways. This current study would deepen our understanding on schizophrenia-related changes of WM integrity, their relationship to clinical behaviors, as well as the DNAm modification underlying the altered WM microstructure in schizophrenia individuals.

## Methods

### Participants

A total of 42 drug-naive patients with first-episode schizophrenia and 38 gender-, age-, and education-matched healthy volunteers were recruited from October 2012 to January 2014 in Henan Mental Hospital, Xinxiang, China. This dataset was previously used by our group (25). Patients were diagnosed by experienced psychiatrists by using the Structured Clinical Interview for the DSM-IV-TR. The disease duration of all patients was no more than 12 months. Healthy volunteers without history of neurological and psychiatric disorders were screened by using SCID-non-patient edition. Any individual with a history of neurological disorder, severe somatic diseases, or substance abuse was excluded from the study.

The protocol of this study was approved by the Ethics Committee of Henan Mental Hospital and Second Xiangya Hospital of Central South University. All procedures followed were in accordance with the Helsinki Declaration of 1975, as revised in 2008 (5). All subjects agreed to participate in this study and gave written informed consent. Four patients withdrew from the follow-up MRI scans. This study was listed on the Chinese Clinical Trial Registry (Registration Number: ChiCTR1800014844).

### Psychopathological Assessments

The severity of all 42 patients' symptoms was assessed on the day of MRI scans by using the Positive and Negative Syndrome Scale (PANSS) (26), which included the positive (PANSS-P, items P1–P7), negative (PANSSN, items N1–N7), general psychopathology (PANSS-G, items G1–G16), and PANSS total (PANSS-T, all 30 items) symptoms.

### Neurocognitive Function Assessments

Participants' immediate memory, working memory, psychomotor speed, visual-motor coordination, and executive function were assessed by using the Digit Span Test (DST, Forward and Backward), and Trail-Making Test (TMT, A/B). DST includes Forward (for immediate memory evaluation) and Backward (for working memory assessment) sections (27, 28). In the DST-Forward section, subjects repeated the numbers as read to them by the experienced examiner. In the DST-Backward section, they were asked to reverse the numbers read to them. The participants' score was the sum of the correctly recalled numbers, respectively, in each section. Trail Making Test (TMT) was used to test psychomotor speed, visual-motor coordination, and executive function (28–31). This test includes section A and B. Psychomotor speed, visual-motor coordination are involved in both TMT-A and TMT-B performance, while executive function, such as set-switching abilities and inhibition control, plays a specific role in completing TMT-B (28–31). In section A, the subjects were instructed to draw lines with pencils to connect the numbers 1–25 in order which were randomly scattered on a standardized page. Similarly, in section B, the participants were asked to connect numbers (1–13) and letters (A–L) in order of alternating numbers and letters. Scores of this test were the time (seconds) taken to complete each section. A maximum time allowed was 300 s.

### Imaging Data Acquisition

Data were scanned on the 3.0T Siemens MRI scanner (Verio) at the Magnetic Imaging Centre of the Henan Mental Hospital. A standard 16-channel head coil was used. T1-weighted images were acquired sagittally by using a spoiled gradient echo pulse sequence with the following parameters: repetition time, 1900 ms; echo time, 2.52 ms; flip angle, 9°; field of view, 250 mm × 250 mm; slice thickness, 1.0 mm; slice gap, 0 mm; and number of slices, 176. DTI images were acquired by using single-shot spin-echo EPI (SS-SE EPI) sequence: repetition time, 7500 ms; echo time, 93 ms; field of view, 240 mm × 240 mm; slice thickness, 3.0 mm, and b, 1000 s/mm^2^ along 64 directions.

### Image Pre-processing and VBA of FA Images

For DTI data, we corrected the distortions caused by eddy currents and removed the head motions by using FSL 5.0 software (http://www.fmrib.ox.ac.uk/fsl), and then calculated the three-dimensional maps and the FA maps of the diffusion tensor by using Diffusion Toolkit software (TrackVis.org). By utilizing SPM8 package (https://www.fil.ion.ucl.ac.uk/spm/), T1-weighted images of each subject were co-registered to the subject's non-diffusion-weighted image (*b* = 0 s/mm^2^), and then normalized to the Montreal Neurological Institute (MNI) space. Parameters from the above transformation were applied to each subject's FA images, and then the volume was resampled into a voxel size of 3×3×3 mm. Each normalized FA image was spatially smoothed by an 8×8×8 mm full width at half maximum Gaussian kernel.

We used SPM8 to perform the general linear regression analysis on the normalized FA images between the two groups in a voxel-based manner, controlling for age, gender, level of education, and head motion. The significant threshold value was set at *P* < 0.05 (Gaussian random field, GRF corrected, voxel-level *P* < 0.01, cluster-level *P* < 0.05). A “difference map” was calculated from the patients compared with healthy controls. We also calculate Cohen's *d* effect sizes for schizophrenia patients vs. healthy controls.

### SVM Analysis

**Figure 1:**
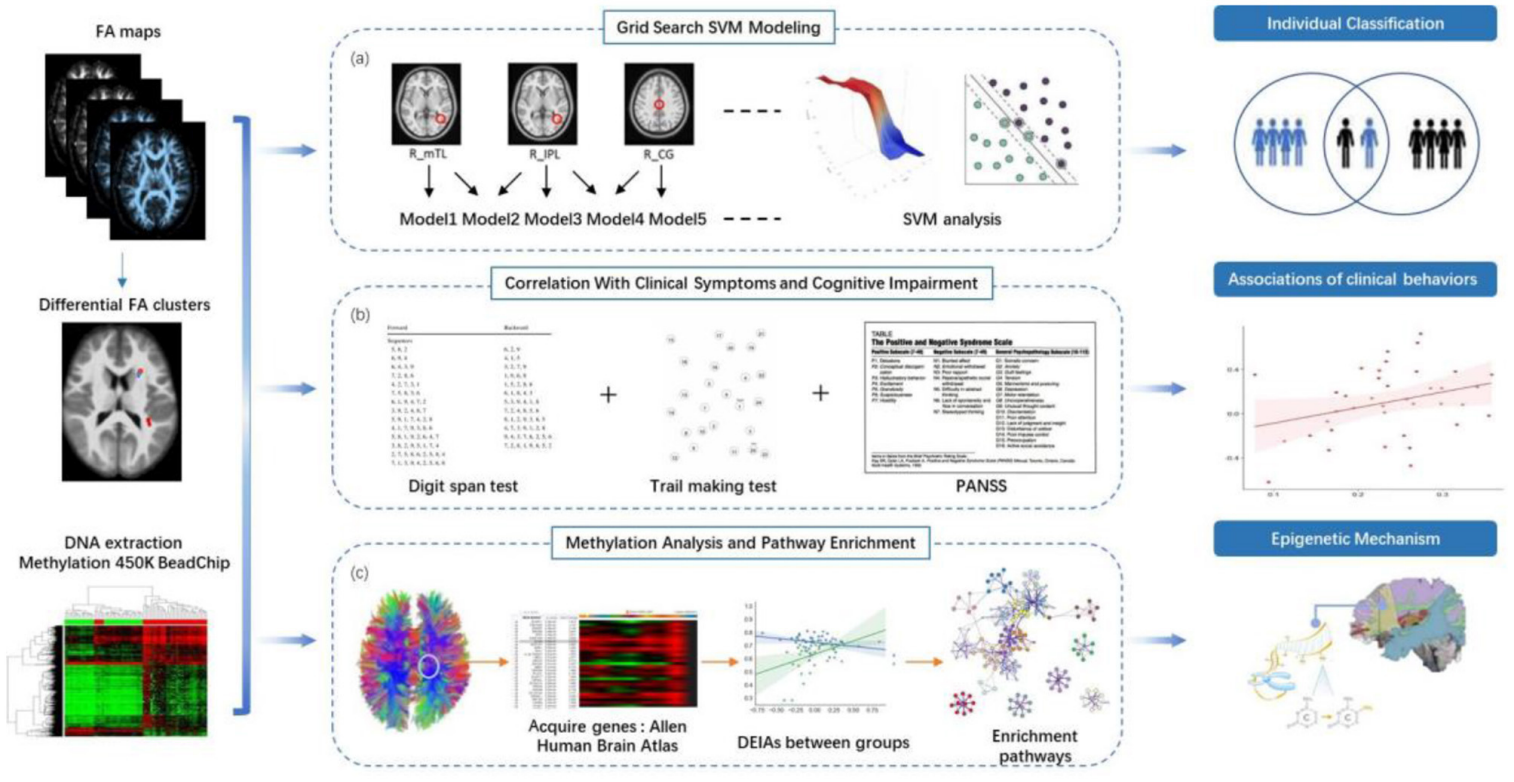
Study overview. **(a)** Between-group comparison of FA and SVM analysis. We identified clusters with between-group differences of FA, listed all the non-repeated combinations according to permutation and combination, and further used support vector machine (SVM) to identify combinations that could distinguish schizophrenia patients from healthy controls accurately. **(b)** Stepwise multiple linear regression was used to compute the correlations between FA values with between-group differences and and clinical behaviors. **(c)** We acquired genes differentially expressed in brain regions with abnormal FA values from the Allen Human Brain Atlas (http://human.brain-map.org/). Methylation levels at the CpG sites for these genes were obtained from the data of this current study. We used primary component analysis (PCA) to visualize the pattern of variability in the gene's methylation status. We analyzed the associations between the component values of the DNA methylation components and FA values with significant between-group differences in both patient and control groups. Z-test was utilized to compare the differential epigenetic-imaging associations (DEIAs) between the two groups. We performed enrichment analysis on Metascape website to find the significant pathways associated with the genes consisting of the DNA methylation components from the DEIAs.

After finding clusters with between-group differences, we then listed all the non-repeated combinations according to permutation and combination, and further used SVM to identify combinations that could distinguish schizophrenia patients from healthy controls accurately. The SVM classifier with Gaussian radial basis function kernel was constructed using the R package “e1071” (https://CRAN.R-project.org/package=e1071). The parameters of the classifier were optimized based on the “leave one out” cross-validation method. Accuracy, sensitivity, and specificity of each combination were calculated. The above process is shown in Figure 1A.

### Associations of Abnormal FA and Clinical Behaviors

The FA values of the “difference map” were extracted for stepwise multiple linear regression (SMLR) analysis to compute the associations between abnormal FA values and clinical behaviors (Figure 1B). The independent variables were the FA values with between-group differences, and the dependent variables were scores on PANSS or cognitive tests. We used the Kolmogorov-Smirnov test to check the normality distribution of dependent variables and the residuals in each equation model of the SMLR analysis.

### Genomic DNAm Data Processing

Among the participants of this study, 38 patients and 38 controls provided whole blood samples. Whole genome methylation status was then examined in the above 76 samples (Figure 1C). This dataset has been previously used by our group (32).

### DNA Extraction

DNA was isolated using QIAamp DNA Blood Mini Kit (Qiagen; Germantown, MD). The A260/A280 ratio measured by utilizing a NanoDrop (Thermo Scientific) was used to assess DNA purity. Agarose gel electrophoresis was used to check DNA quality.

### Bisulfite Conversion and Illumina 450K Genechip Analysis

Genome-wide DNAm was treated with sodium bisulfite and then quantified, respectively, by using the EZ DNAm kit (Zymo Research; Irvine, CA) and Infinium® Human Methylation 450K BeadChip (Illumina Inc.; San Diego, CA). According to the recommendations of EZ DNAm Kit manufacturer (Zymo Research; Irvine, CA), 1 μg DNA was utilized for sodium bisulfite-treated assay. Detailed information about the Illumina 450K Genechip analysis, the QC controls of Genechip assay, microarray data processing was shown in Supplementary Material and section Methods.

### Acquiring Gene of Interest and Principal Component Analysis

We acquired the name of genes differentially expressed in the observed brain regions with abnormal FA through a web search on the Allen Human Brain Atlas (24) (http://human.brain-map.org/) (see Figure 1C). Methylation levels at the CpG sites for these genes were obtained from the genomic DNAm data of this current study. In order to incorporate biological knowledge, we computed the average value of methylation sites within a gene. Then we performed primary component analysis (PCA) to visualize the pattern of variability in genes' methylation status. Component values, i.e., contribution values, for the detected DNAm primary components were utilized for the following epigenetic-imaging association analysis.

### Calculation for the Differential Epigenetic-Imaging Associations Between Case and Control Groups

Pearson correlation was used to compute the correlations between the abnormal FA values and the component values of the above-detected DNAm principal components, respectively, for both patient and control groups (the significance level was set at *P* < 0.05 with FDR correction). If there were significant correlations in either patient or control group, we would then perform a Z-test to compare the differential epigenetic-imaging associations (DEIAs) between the two groups (Figure 1C).

We used Bootstrap Confidence Intervals (33) to produce a 95% confidence interval (95% CI) around the estimate of Z value differences, which represented the between-group differences of the correlation coefficient in the DEIAs analysis. Specifically, we randomly selected 90% of the samples, respectively, from patients and healthy controls for 1,000 times. The interval is produced by the interval between the 2.5-th and 97.5-th percentile point of the bootstrap distribution of Z values.

### Enrichment Pathways for Genes in the DEIAs

After finding the between-group DEIAs, we performed enrichment analysis for the genes consisting of the DNAm components by using the Metascape (http://david.abcc.ncifcrf.gov/) (34), an online tool that provides automated online meta-analysis to obtain either unique or common enriched pathways in 40 independent knowledge bases. The gene's name was input into the Metascape website, and the enrichment terms were filtered to those pathways that met a *P*-value < 0.05 with FDR correction.

### Statistical Analysis

SPSS version 17.0 (SPSS Inc., USA) was conducted to compare the between-group differences of demographic and cognitive function scores in patients and healthy volunteers by using the independent two-sample *t*-test as well as chi-squared tests for continuous and categorical variables, respectively. The significance level was set at *P* < 0.05.

The stepwise multiple linear regression (SMLR) was used to investigate the effect of FA values with significant between-group differences on patients' psychopathological symptoms and cognitive impairment. The significance level was set at *P* < 0.05, with FDR correction. We add the information about the FDR correction to the Statistical Analysis in Supplementary Material.

## Results

### Demographics and Clinical Behaviors

No significant differences were found in the between-group comparisons of the demographic characteristics of healthy volunteers and patients (*P*s > 0.05; Supplementary Table S1). Patients' positive (25.60 ± 3.75), general psychopathology (48.14 ± 6.46), negative symptoms (18.17 ± 5.21), and total symptoms (91.90 ± 11.23) were assessed.

Patients' performance on DST_forward, DST_backward, TMT_A, and TMT_B (*P*s < 0.001, corrected by FDR) was significantly poorer than that of healthy controls (see Supplementary Table S2).

### Between-Group Comparison of FA

**Figure 2:**
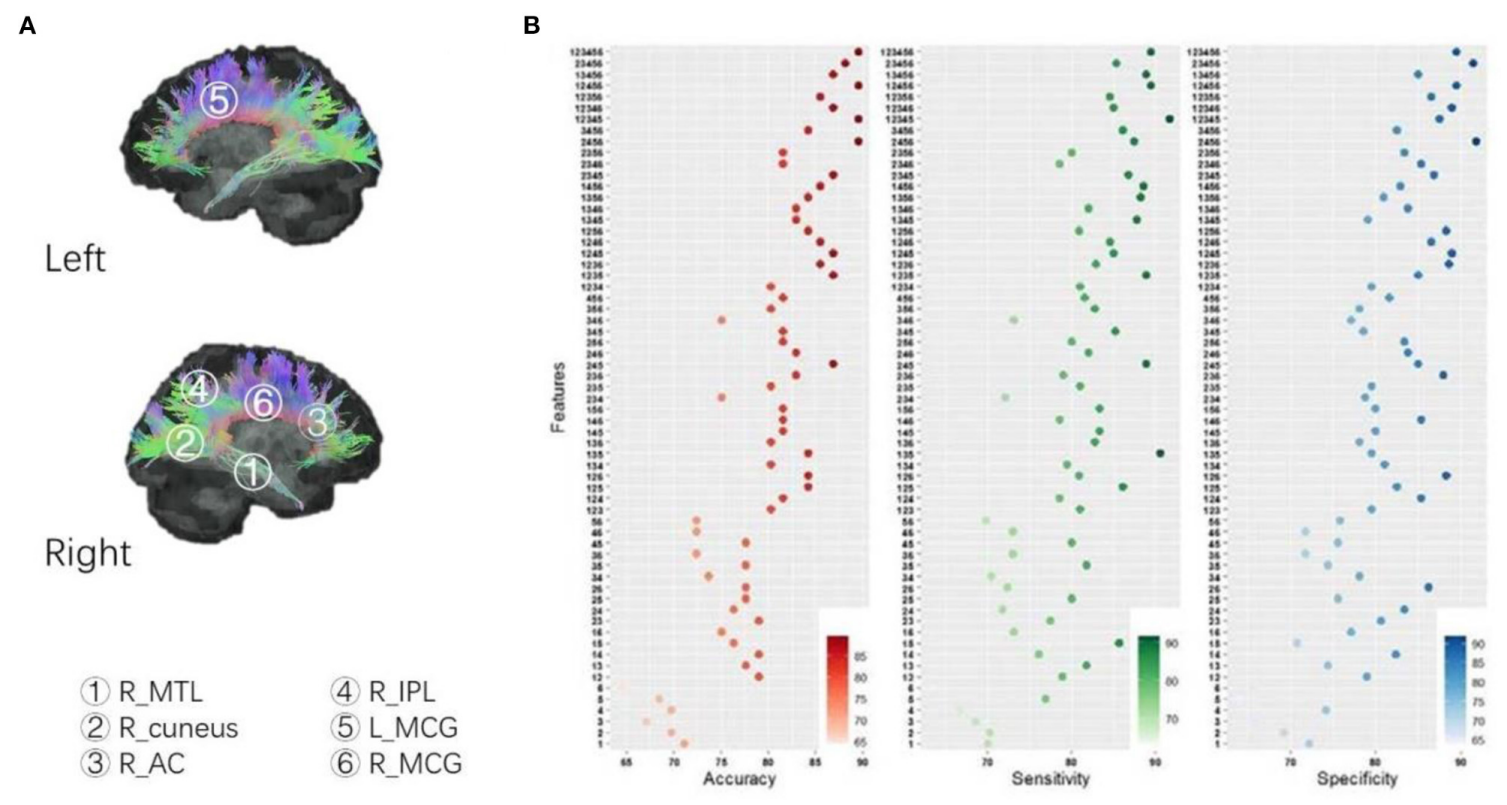
**(A)** Between-group comparison of FA; **(B)** SVM analysis: identify combinations accurately distinguishing patients from controls. According to the permutations and combinations, the six clusters with between-group differences of FA (patients vs. controls) constitute 63 non-repeated combinations, in which four combinations in the table showed maximum accuracy, a combination (cluster 1+2+3+4+5) showed maximum sensitivity, and a combination (cluster 2+4+5+6) demonstrated maximum specificity. Cluster 1: R_MTL, right middle temporal lobe; Cluster 2: R_cuneus, right cuneus; Cluster 3: R_AC; Cluster 4: R_IPL, right inferior parietal lobe; Cluster 5: L_MCG, left middle cingulate; Cluster 6: R_MCG, right middle cingulate.

The FA images were compared at the voxel-wise level by using a two-sample *t*-test between healthy controls and patients. Significant decreases in the FA values were detected to be located in the right middle temporal lobe (R_MTL) WM (*P* < 0.01 with GRF correction, Cohen's *d* = 1.238), R_cuneus WM (*P* < 0.01 with GRF correction, Cohen's *d* = 0.956), right anterior cingulate (R_AC) WM (*P* < 0.01 with GRF correction, Cohen's *d* = 0.988), and right inferior parietal lobe (R_IPL) WM (*P* < 0.01 with GRF correction, Cohen's *d* = 0.871) in first-episode schizophrenia patients, while a significant increase in the left middle cingulate (L_MCG) WM (*P* < 0.01 with GRF correction, Cohen's *d* = 0.780) and right middle cingulate (R_MCG) WM (*P* < 0.01 with GRF correction, Cohen's *d* = 0.978) (see Figure 2A). MNI locations of the above six clusters were showed in Supplementary Table S3.

### SVM Analysis: Identify Combinations Accurately Distinguishing Patients From Controls

According to the permutations and combinations, the above 6 clusters with between-group differences (patients vs. controls) in FA values constitute 63 non-repeated combinations. We performed SVM to evaluate the accuracy, sensitivity, and specificity of combinations for abnormal FA values in distinguishing patients from healthy volunteers.

We uncovered a trend that the more clusters that were combined, the higher the accuracy of the abnormal FA values in discriminating patients from healthy volunteers (Figure 2B). We also found that abnormal FA values in four combinations had 89.47% accuracy, which was higher than the other 59 combinations (see Figure 2B). The minimal, median, and maximum of accuracy, sensitivity, and specificity of all 63 combinations are detailed in Supplementary Table S4.

### Abnormal FA Values and Correlations With Symptoms and Cognitive Function in Patient and Control Groups

We performed SMLR in a patient group to determine the relationship of abnormal FA values with clinical symptoms as well as cognitive abnormalities. The assessment of the normality of dependent variables and the residuals of each equation model was showed in the Supplementary Table S5.

**Figure 3:**
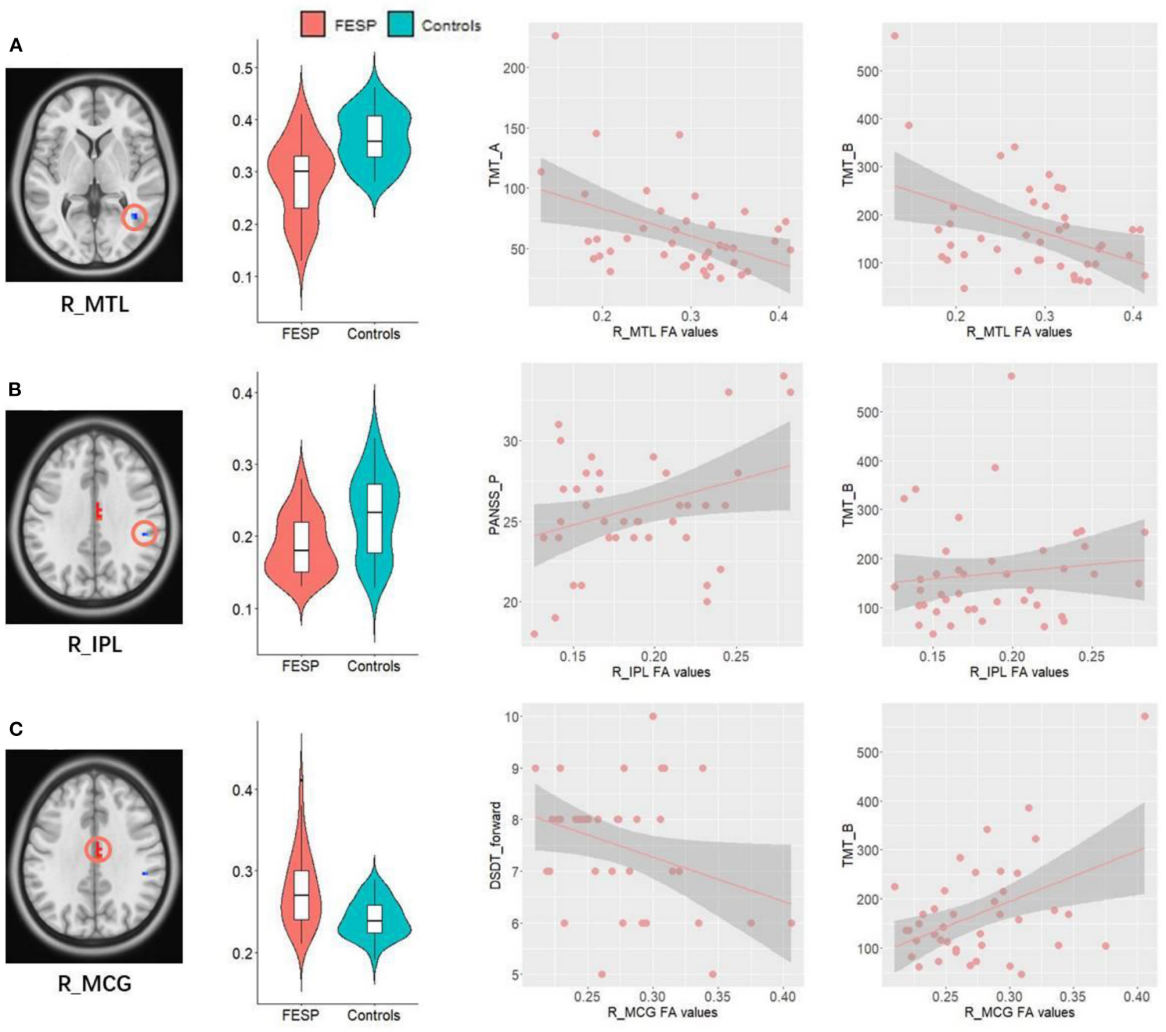
Correlations between abnormal FA values and clinical behaviors. **(A)** Patients' abnormally decreased FA values in R_MTL were significantly negatively associated with their increased time spending in performing TMT_A and TMT_B. **(B)** Decreased FA values in the R_IPL WM were positively correlated with the severity of PANSS_P, as well as increased time spending on completing TMT_B. **(C)** Patients' abnormally increased FA values in R_MCG showed significantly positive correlations with increase of time in completing TMT_B, as well as a negative relationship with number of DST_forward. R, right; L, left; MTL, middle temporal lobe; IPL, inferior parietal lobe; MCG, middle cingulate; TMT, Trail-Making Test; DST, Digit Span Test; PANSS_P, Positive and Negative Syndrome Scale_positive symptoms.

Patients' abnormally decreased FA values in R_MTL were significantly negatively associated with their increased time spending in performing TMT_A (*P* = 0.0055, *R*^2^ of the regression model = 0.181, *P* of the regression model with FDR corrected = 0.0222, Figure 3A) and TMT_B (*P* = 0.0183, *R*^2^ of the regression model = 0.381, *P* of the regression model with FDR corrected = 0.0035, Figure 3A).

Decreased FA values in the R_IPL WM were positively correlated with the severity of PANSS_P (*P* = 0.041, *R*^2^ of the regression model = 0.103, P of the regression model with FDR corrected = 0.0843, Figure 3B), as well as increased time spending on completing TMT_B (*P* = 0.0398, *R*^2^ of the regression model = 0.381, *P* of the regression model with FDR corrected = 0.0035, Figure 3B), indicating that the lower the FA values in the R_IPL, the milder the executive function impairments and positive symptoms in patients. Patients' abnormally increased FA values in R_MCG showed significantly positive correlations with increase of time in completing TMT_B (*P* = 0.0015, *R*^2^ of the regression model = 0.381, P of the regression model with FDR corrected = 0.0035, Figure 3C), as well as a negative relationship with number of DST_forward (*P* = 0.042, *R*^2^ of the regression model = 0.101, *P* of the regression model with FDR corrected = 0.0843, Figure 3C).

### DEIAs Between Groups and Enrichment Analysis

Abnormal FA values of voxels with significant between-group differences, i.e., voxels in the R_MTL, R_cuneus, R_AC, R_IPL, and bilateral MCG, were mainly located in the cingulum tracts due to previous reports (35). We then acquired genes differentially expressed in cingulum through searching the web of the Allen Human Brain Atlas (http://human.brain-map.org/). We selected the option “Differential Search” and checked “cgb cingulum bundle” as the “Target Structure(s)” and “Brain” as the “Contrast Structure(s),” and then downloaded the data containing a total of 22,487 genes (38,685 corresponding probes) differentially expressed in the cingulum bundle as compared with the whole brain. Methylation levels at the CpG sites for the 22,487 genes were obtained from the data of our current study. The average value of methylation sites within a gene was computed to represent the gene's methylation level, and then PCA was performed to visualize the pattern of variability in all the 22,487 genes' methylation status. After PCA, 16 DNAm primary components were detected. We analyzed the associations between the component values of the 16 DNAm components and FA values of the 6 clusters with significant between-group differences for both patient and control groups.

**Figure 4:**
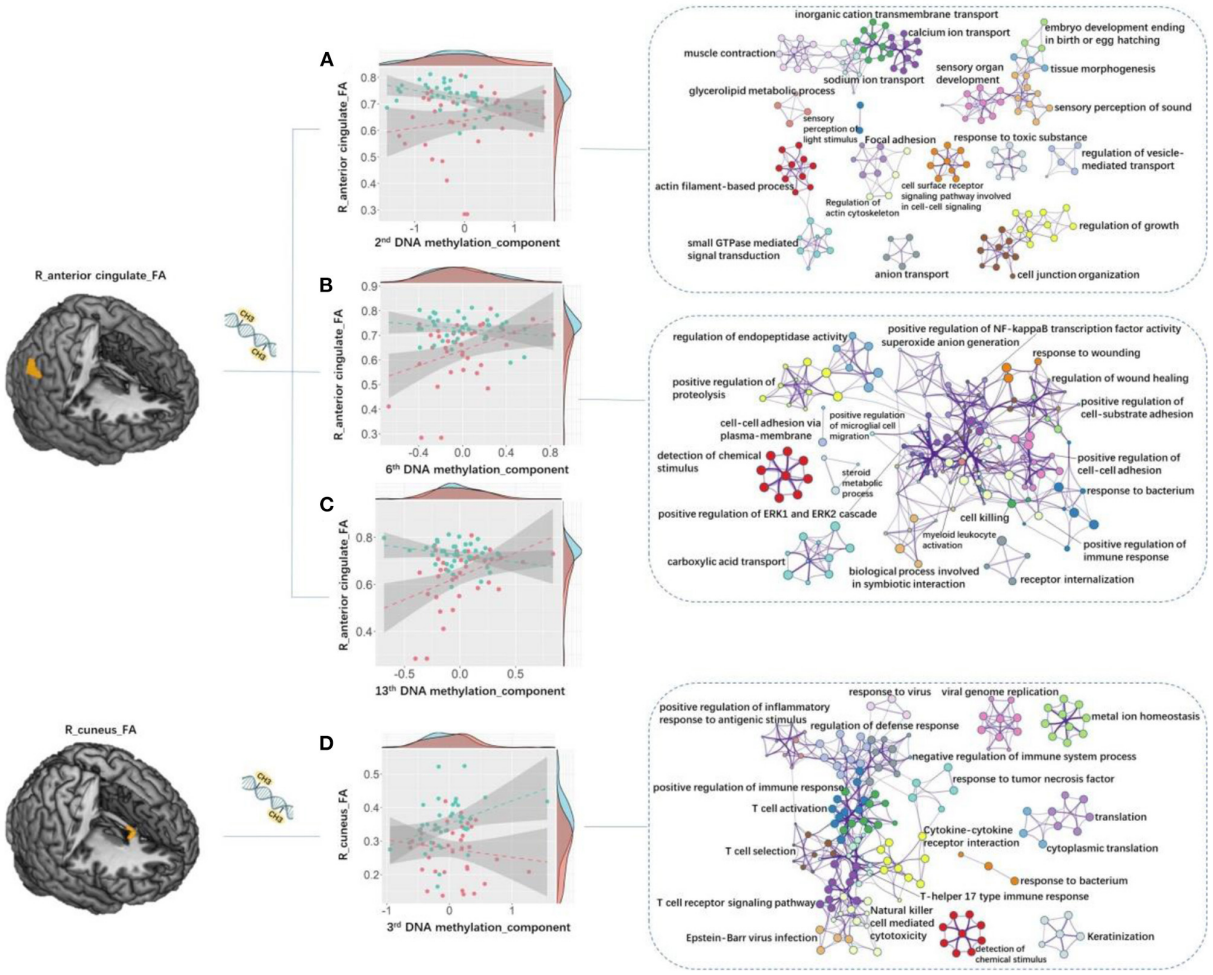
Between-group DEIAs. By computing the DEIAs between the two groups, we found that: **(A)** the association between the 2nd DNA methylation component and R_AC_FA showed significant between-group difference, and the “actin filament-based process” and “sensory perception of light stimulus” pathways discovered for genes in the 2nd DNAm component showed relatively higher Metascape values out of all other processes and pathways; **(B)** the association between the 6th DNA methylation component and R_AC_FA was significantly different, and genes in the 6th DNAm component enriched in the “detection of chemical stimulus” process demonstrated the highest Metascape value compared with other processes or pathways; **(C)** the association between the 13th component and R_AC_FA showed significant difference; **(D)** the association between the 3rd component and R_ Cuneus_FA was significant, and genes in the 3rd DNAm component primarily enriched in cognitive and immunologic processes and pathways such as “detection of chemical stimulus involved in sensory perception”, “T cell activation”, “positive regulation of immune response”, and “T cell receptor signaling pathway”. Each circle node represents an enrichment term. Nodes with the same color belong to the same term (i.e., cluster). The circle node size is equal to the input genes number included in that cluster. DEIAs, differential epigenetic-imaging associations; R, right; L, left; AC, anterior cingulate; FA, fractional anisotropy.

By computing the DEIAs between the two groups, we found that the associations between the R_AC_FA and the 2nd DNAm component (*Z* = −3.61, *P* = 0.0049 corrected by FDR, [95% CI −2.759 ~ −0.4.224], Figure 4A), R_AC_FA and the 6th DNAm component (*Z* = −3.73, *P* = 0.00451 corrected by FDR, [95% CI −2.441 ~ −0.4.429], Figure 4B), R_AC_FA and the 13th DNAm component (*Z* = −4.32, *P* = 0.000741 corrected by FDR, [95% CI −3.137 ~ −0.4.717], Figure 4C), as well as R_Cuneus_FA and the 3rd DNAm component (*Z* = 3.17, *P* = 0.0186 corrected by FDR, [95% CI 2.270 ~ 3.738], Figure 4D), showed significant differences between patient and healthy control groups.

We then used Metascape to align the Kyoto Encyclopedia of Genes and Genomes (KEGG) pathways and gene ontology (GO) biological processes, respectively, for the gene lists of 2nd, 3rd, 6th, and 13th DNAm components. We corrected for enrichment terms with *P*_FDR_ < 0.05 and discarded discrete enrichment. Genes in the 4 DNAm components were significantly enriched in neuronal, immunologic, and cognitive processes and pathways. In particular, the “actin filament-based process” and “sensory perception of light stimulus” pathways discovered for genes in the 2nd DNAm component showed relatively higher Metascape values out of all other processes and pathways (Figure 4A; Supplementary Table S6). Genes in the 3rd DNAm component primarily enriched in cognitive and immunologic processes and pathways such as “detection of chemical stimulus involved in sensory perception,” “T cell activation,” “positive regulation of immune response,” and “T cell receptor signaling pathway” (Figure 4D; Supplementary Table S6). Genes in the 6th DNAm component enriched in the “detection of chemical stimulus” process demonstrated the highest Metascape value compared with other processes or pathways (Figure 4B; Supplementary Table S6).

## Discussion

This study combined DTI and peripheral blood genomic DNAm and aimed to investigate schizophrenia-related changes of WM microstructures, their relationship to psychopathological symptoms and cognitive impairments, as well as the epigenetic basis underlying the altered WM integrity in this disease.

As hypothesized, patients had significantly decreased FA located in the R_MTL, R_cuneus, R_AC, and R_IPL WM, while a significant increase in bilateral MCG WM. These results on the one hand suggested that schizophrenia may be a whole-brain WM disorder, but not confined to the abnormality of a specific brain region, on the other hand verified the regional variation in severity with prefrontal, temporal, parietal, and cingulate WM being more affected. The observed six clusters with abnormal FA values have been previously reported to be connected by cingulate tract, which is the most important fiber bundle within the limbic system (35). The cingulate bundle underlies and provides the cingulate gyrus with connections from and to the anterior, middle, and posterior portions of Broca's “grand limbic lobe” (35). However, abnormal FA values in 6 brain regions may not be specific to schizophrenia, given that the anomalies have been also detected in other neuropsychiatric disorders (36, 37). In the SVM analysis, the FA value of any one of the 6 clusters alone was too low to distinguish patients from controls; this phenomenon is highly consistent with our inference that schizophrenia may not be confined to the abnormality of a specific brain region. Furthermore, we found that abnormal FA values in four combinations had 89.47% accuracy, which was higher than that of the other 59 combinations coupling each abnormal ReHo value in the regions. This finding might help us distinguish schizophrenia patients from healthy population.

This study found that FA values of both middle (R_MCG) and posterior (R-IPL and R_MTL) cingulate bundles were related to patients' impaired executive function. These findings are consistent with previous evidence linking cingulum fasciculus integrity to deficits in executive functions in schizophrenia patients (4, 38). Except executive function, we found that the integrity of distinct portions of the cingulate bundle was involved in different neurocognitive domains or psychopathological symptoms. Specifically, the FA values of R_IPL and R_MTL WM, consisting of the posterior part of cingulum, were associated with patients' positive symptoms, psychomotor speed, and visual-motor coordination; integrity of R_MCG (median part) was related to patients' immediate memory. The posterior portion of cingulate bundle is strongly interconnected with sensory-related cortex such as the parietal, medial temporal, and orbitofrontal cortex; previous studies have linked it with hallucination, delusion, and cognitive function such as top-down control of eye movements and visual attention (39, 40). As to the mid-cingulate, its resting-state functional connectivity abnormalities have been reported to be involved in the schizophrenia pathology (41). This study first showed the effect of median cingulate WM anomalies on immediate memory impairments. The anterior part of cingulum, mainly interconnected with AC, prefrontal cortex, and part of the subcortical structures, was more often reported to be related to positive symptoms than the posterior part (4, 35), although we did not detect the associations in this study. While acknowledging the fact that this study did not track the cingulate bundle alone, these current findings support the point that the cingulate bundle may be a conglomeration of anatomically connected yet functionally different sub-connections. Particularly worth mentioning is that the decrease of FA in the R_IPL WM was positively correlated with PANSS_P scores, as well as patients' time consuming when completing TMT_B, indicating that the more severe the WM damage in the R_IPL, the less severe the executive function impairments and positive symptoms. We speculate that WM integrity of the R_IPL may play a compensatory role in the early phase of this disease, although the actual functional significance of the so-called compensatory effect remains to be verified.

Schizophrenia is a heterogeneous, heritable, and serious mental disorder. Limited success has been seen in exploring causal genes for this disease from numerous conventional studies. Investigating the epigenetic effect on a brain-based association of schizophrenia—instead of disease pathology status—decreases symptom heterogeneity. In addition, it allowed us to explore the effect of a potential risk factor on the continuously distributed brain-imaging intermediate phenotypes in individuals with schizophrenia as well as in healthy volunteers (42). This study demonstrated the between-group differences of the relationships between abnormal FA values and DNAm components, which suggested the abnormal couple patterns of WM integrity-DNAm in schizophrenia. We demonstrated that the enriched pathways or biological processes in the 2nd, 3rd, and 6th DNAm components were primarily related to neuron axon, neurocognition, and immune process. Such findings agree with the belief that the etiology and development of schizophrenia, such a complex disease, may be the result of simultaneous disturbance of multiple pathways or biological processes. This study, to our knowledge, demonstrated first the associations between DNAm and WM microstructure abnormalities in schizophrenia, and implies that impairments of WM integrity in schizophrenia may be epigenetically regulated.

Pathways in the 2nd component were mainly related to neuron axon and neurocognition processes, e.g., the “actin filament-based process” which may participate in the initiation and branching of neuron axon (43), as well as the “sensory perception of light stimulus” process and other cognitive processes such as “sensory organ development” and “sensory perception of sound” (Figure 4A; Supplementary Table S6). Interestingly, the neurodevelopmental model also highlighted the importance of actin in the genesis of schizophrenia: chronic actin depolymerization generated in the process of prenatal viral infection may change the cell structure and result in dysregulation of multiple signaling systems observed in schizophrenia (2). This finding supports the widely accepted neurodevelopmental anomalies and cognitive impairments in schizophrenia (2, 44, 45). The “detection of chemical stimulus” process of the 6th DNAm components, also relating to neurocognitive process, showed the highest Metascape values out of all other biological processes (Figure 4B; Supplementary Table S6). This process involves a series of neurocognitive events in which a chemical stimulus is received and then converted into molecular signaling as part of sensory perception.

Among the top 20 biological processes and pathways of the 3rd DNA components, 13 were involved in or associated with the immune system (Figure 4D; Supplementary Table S6). Dysregulations or abnormalities of the immune-related processes in schizophrenia have been previously observed (46). This current finding correlated WM deficits to DNAm abnormalities of immune-related genes, and supports the neurodevelopmental hypothesis for schizophrenia, which suggested that the abnormal maternal cytokines, such as TNF-a, IL-1b, or IL-6 induced by prenatal infection, may regulate the offspring's brain development process (2). Studies in mice, human, and non-human primates also suggested a link between immune processes and anomalies of anatomical connectivity integrity in the brain of individuals with schizophrenia, older adults with cognitive impairment, as well as individuals with Alzheimer's disease and major depressive disorder (47–50). An epigenetic-neuroimaging study demonstrated that both DNAm and global efficiency of functional brain network altered after cognitive remediation program in schizophrenia individuals (51). Their work also revealed the positive associations between changes of global efficiency and methylation of *RELN* and *BDNF* genes (51), which provided an integrated macro- and microscopic perspective of the neuroplastic mechanisms underlying cognitive impairments in schizophrenic individuals, and linked the associations among DNAm, brain structural connectivity, and cognitive function in this disease. Based on this evidence, we proposed that DNAm of genes enriched in neuronal, immunologic, and cognitive processes observed in this study may serve as the basis in the effect of WM deficits on clinical behaviors in schizophrenia.

Several limitations need to be considered. The first is the small sample size in this neuroimaging-epigenetic study, although this is the first study creatively exploring both DTI and DNAm datasets acquired from the same sample of schizophrenia patients. Second, the DNAm status was derived from the peripheral blood cells, rather than brain cingulum fasciculus, which limits the ability to understand the epigenetic basis in the effect of WM integrity deficits on clinical behaviors, despite the proposed “mirror” pattern that the DNAm state in the blood is related to the corresponding disease-causing regions in the brain (23). Third, the DNA methylation of whole blood cells represents a mixture of heterogeneous cells (52), and it therefore was not able to factor out the potential influence of DNAm variation across different cell types. Fourth, this study did not control intelligence quotient (IQ), an important factor for assessing cognitive function in patients with schizophrenia, which limits the ecological validity of the neuropsychological methods used in this study.

In conclusion, there have been only a few neuroimaging studies simultaneously examining the WM integrity alterations related to the severity of psychopathological symptoms and cognitive impairments, as well as the epigenetic basis underlying the abnormal FA in the same schizophrenia individuals. Our findings suggest that schizophrenia may not cause widespread neuropathological changes affecting all WM, but subtle alterations affecting local cingulum WM, which may relate to positive symptoms and cognitive impairments such as executive function, working memory, immediate memory, psychomotor speed, and visual-motor coordination. This imaging-epigenetics study revealed for the first time that DNAm of genes enriched in neuronal, immunologic, and cognitive processes may serve as the basis in the effect of WM deficits on clinical behaviors in schizophrenia.

## Data Availability Statement

The datasets presented in this study can be found in online repositories. The names of the repository/repositories and accession number(s) can be found at: ArrayExpress, E-MTAB-11078. Further inquiries can be directed to Maolin Hu, humaolin@whu.edu.cn.

## Ethics Statement

The studies involving human participants were reviewed and approved by The Second Xiangya Hospital of Central South University. The patients/participants provided their written informed consent to participate in this study.

## Author Contributions

XZon, XZou, HC, JZhe, and MH conceived and designed the study. QZ, CH, XH, JZha, GW, LL, and DS performed the investigation and data analysis. XZon, JZhe, and MH contributed to the interpretation of the data. XZon, QZ, and CH wrote the manuscript. All authors contributed to the article and approved the submitted version.

## Funding

This study was supported by grants from the National Natural Science Foundation of China (81901357) and the National Key R&D Program of China (2018YFC1314600).

## Conflict of Interest

The authors declare that the research was conducted in the absence of any commercial or financial relationships that could be construed as a potential conflict of interest.

## Publisher's Note

All claims expressed in this article are solely those of the authors and do not necessarily represent those of their affiliated organizations, or those of the publisher, the editors and the reviewers. Any product that may be evaluated in this article, or claim that may be made by its manufacturer, is not guaranteed or endorsed by the publisher.
