# Supplementary material for: DNA Methylation Basis in the Effect of White Matter Integrity Deficits on Cognitive Impairments and Psychopathological Symptoms in Drug-Naive First-Episode Schizophrenia
Source: Front Psychiatry. 2021 Dec 13;12:777407. doi: 10.3389/fpsyt.2021.777407 (PMC8710603; doi:10.3389/fpsyt.2021.777407)
Supplement: Supplementary file 1 [file Data_Sheet_1.PDF]

DNA methylation basis in the effect of white matter integrity deficits on cognitive impairments and psychopathological symptoms in drug-naive first-episode schizophrenia

Xiaofen Zong[1,2 †], Qinran Zhang[3,4 †], Changchun He[5+], Xinyue Huang[5], Jiangbo Zhang[5], Gaohua Wang[1], Luxian Lv[6], Deen Sang[6], Xiufen Zou[3,4]*, Huafu Chen[5]*, Junjie Zheng[7,8]* and Maolin Hu[1,2]*

[1] Department of Psychiatry, Renmin Hospital of Wuhan University, Wuhan, China,
[2] Department of Psychiatry, the Second Xiangya Hospital, Central South University, Changsha, China,
[3] School of Mathematics and Statistics, Wuhan University, Wuhan, China,
[4] Hubei Key Laboratory of Computational Science, Wuhan University, Wuhan, China,
[5] High-Field Magnetic Resonance Brain Imaging Key Laboratory of Sichuan Province, School of Life Science and Technology, University of Electronic Science and Technology of China, Chengdu, China,
[6] Department of Psychiatry, Henan Mental Hospital, the Second Affiliated Hospital of Xinxiang Medical University, Xinxiang, China,
[7] Early Intervention Unit, Department of Psychiatry, Affiliated Nanjing Brain Hospital, Nanjing Medical University, Nanjing, China,
[8] Functional Brain Imaging Institute of Nanjing Medical University, Nanjing, China

*Correspondence: Maolin Hu, humaolin@whu.edu.cn; Xiufen Zou, xfzou@whu.edu.cn; Huafu Chen, chenhf@uestc.edu.cn; Junjie Zheng, zjj5270@163.com

[†] These authors contributed equally to this work.

SUPPLEMENTARY MATERIALS AND METHODS

Illumina 450K Genechip Analysis

We randomly distributed the two groups to different arrays in order to control the batch effects. DNA methylation level was measured using the Illumina Infinium HD Methylation Assay (Illumina) according to the manufacturer's instructions. We downloaded the manifest files of each array in advance and imaged the BeadChips using an Illumina Scan.

The QC Controls of Genechip Assay

The QC controls of all samples from the two groups were tested by using the methylation module of the GenomeStudio software (v1.9). The controls included sample-dependent (bisulfite conversion, stringency, non-polymorphic and negative controls), as well as sample-independent controls（staining, extension, target removal and hybridization controls). We qualified the above controls according to the Illumina’s instruction.

Illumina 450K Microarray Data Processing

Raw data of Illumina array was processed by using the methylation module of the GenomeStudio (v1.9) based on default parameters, which outputed signal intensities and detection P-values of all probes. We then filtered the samples which had more than 10% CpG sites with detected $P > 0.05$ and probes which had more than 25% samples with detected $P > 0.05$. Raw data were normalized by utilizing BMIQ R packages and Lumi including probe type bias and color-bias adjustment. We used Lumi to convert the individual sites for each sample into methylation levels, i.e., β-values, which ranged from 0 to 1 and were computed as the ratio of the DNA methylation signal intensity to the sum of both unmethylated and methylated signals.

Statistical Analysis

When we used the stepwise multiple linear regression (SMLR) to investigate the effect of FA values with significant between-group differences on patients’ psychopathological symptoms (PANSS-T, PANSS-P, PANSS=N, PANSS-G) and cognitive function (DST_forward, DST_backward, TMT_A, and TMT_B), P values of each regression model were corrected with FDR due to multiple (8) regression models we constructed, which are shown below. We set the significance level at $P < 0.05$ with FDR correction.

RESULTS

Table S1 Demographics for Schizophrenia Patients and Healthy Controls

Variable	Patients (n=42)		Controls (n=38)		Analysis[a]	
	M	SD	M	SD	t	P
Age (years)	24.86	4.80	24.76	4.56	0.09	0.929
Education	10.48	2.84	11.05	2.91	0.90	0.373
	Yes	No	Yes	No	χ^2	P
Gender (male)	27	15	25	13	0.02	0.888
Handedness (right)	42	0	38	0		

[a]patients at baseline *vs* healthy controls, two-sample t test.

Table S2 Comparison of Cognitive Function in Patient and Control Group

Variables	Patients at Baseline (n=41)		Controls (n=38)		Analysis[a] (FDR correction)	
	M	SD	M	SD	t	P
DST_forward	7.49	1.19	8.66	1.26	4.256	**<0.001**
DST_backward	4.88	1.40	6.50	1.45	5.062	**<0.001**
TMT_A-time (s)	63.46	38.68	34.69	13.52	-4.343	**<0.001**
TMT_B-time (s)	169.52	103.06	71.82	21.84	-5.724	**<0.001**

Note: Our current study recruited 42 drug-naïve first-episode schizophrenia patients, of which 41 individuals completed cognitive function tests. [a]41 patients *vs* 38 healthy volunteers, two-sample t test. Abbreviation: DST, Digit Span Test; TMT, Trail Making Test.

Table S3 MNI Locations of the Brain Regions with Altered FA in Patients Compared with Controls

Clusters	Peak MNI coordinate region	Normality distribution of FA values[a]		Cluster size (voxels)	Peak intensity	Peak MNI coordinate			Statistical power[b]
		Patients	Controls			x	y	z	

Cluster 1	R_Middle Temporal Lobe	P=0.741	P=0.746	23	-4.1983	39	-60	3	1
Cluster 2	R_Cuneus	P=0.834	P=0.876	20	-4.2639	21	-78	12	0.99992
Cluster 3	R_ Anterior Cingulate Lobe	P=0.291	P=0.372	22	-4.2792	9	9	24	0.99996
Cluster 4	R_ Inferior Parietal Lobe	P=0.436	P=0.720	21	-3.7437	51	-33	33	0.99946
Cluster 5	R_Middle Cingulate	P=0.592	P=0.691	17	3.9991	3	-18	36	0.99676
Cluster 6	L_Middle Cingulate	P=0.779	P=0.968	24	3.7575	-6	6	39	0.99995

Note: P<0.01 (GRF correction). Abbreviations: R, right; FA, Fractional Anisotropy; MNI, Montreal Neurological Institute.

a: We tested the normality distribution using the Kolmogorov-Smirnov test.

b: We calculated the statistical power using the Python package Statsmodels (https://www.statsmodels.org/stable/stats.html).

Table S4 SVM Analysis: the Accuracy, Specificity and Sensitivity for Different Feature Numbers of 63 Combinations

Feature_N	Conbination_N	Accuracy			Specificity			Sensitivity		
		Minimal (%)	Median (%)	Maximum (%)	Minimal (%)	Median (%)	Maximum (%)	Minimal (%)	Median (%)	Maximum (%)
1	6	64.47	69.08	71.05	64.00	67.54	74.19	63.41	69.29	76.92
2	15	72.37	77.63	78.95	70.83	75.76	86.21	69.77	76.19	85.71
3	20	75.00	81.58	86.84	77.14	80.54	88.24	72.09	81.33	90.63
4	15	80.26	84.21	89.47	79.07	85.00	91.67	78.57	85.00	88.89
5	6	85.53	87.50	89.47	85.00	88.19	91.43	84.62	87.13	91.67
6	1	89.47	89.47	89.47	89.47	89.47	89.47	89.47	89.47	89.47

Note: According to the permutations and combinations, the 6 clusters with between-group differences in FA (patients *vs* controls) constitute 63 non-repeated combinations. We used support vector machine (SVM) to identify the combination that could accurately distinguish schizophrenia patients from healthy

controls. We calculated the accuracy, sensitivity and specificity of each the 63 combinations. Feature number "N" represents any "N" clusters. For example, 1 feature represents any 1 cluster; 2 features represent any 2 clusters; and 3 features represent any 3 clusters. Abbreviations: N, numbers.

Table S5 The assessment of the normality distribution of dependent variables and the residuals in each equation model of the SMLR analysis

Equation modes of SMLR analysis	normality of Dependent variables[a]		normality of the residuals[a]	
Clinical symptoms	K-S statistic values	P	K-S statistic values	P
Y: PANSS-T	0.118	0.574	0.121	0.565
Y: PANSS-P	0.137	0.386	0.101	0.776
Y: PANSS-N	0.142	0.346	0.144	0.342
Y: PANSS-G	0.091	0.859	0.084	0.914
Cognitive function	K-S statistic values	P	K-S statistic values	P
Y: DST_forward	0.256	0.007	0.108	0.680
Y: DST_backward	0.196	0.072	0.196	0.073
Y: TMT_A	0.190	0.087	0.119	0.559
Y: TMT_B	0.163	0.197	0.117	0.580

Note: Abbreviations: SMLR, stepwise multiple linear regression; DST, Digit Span Test; TMT, Trail Making Test; PANSS, Positive and Negative Syndrome Scale; PANSS-T, PANSS total symptom scores; PANSS-P, PANSS positive symptom scores; PANSS-N, PANSS negative symptom scores; PANSS-G, general psychopathology symptom scores; K-S, Kolmogorov-Smirnov test; DST, Digit Span Test; TMT, Trail Making Test.

a: We tested the normality distribution using the Kolmogorov-Smirnov test.

Table S6 Enriched Pathways and Biological Processes of the Four DNA Methylation Components in the Differential Epigenetic-imaging Associations Analysis

DNAm Component	Number of Genes in the components	Number of methylated CpG sites in the components	Term	Category （GO/KEGG）	Description	Count	%	Log10(P)	Log10(q)
2nd components	1933	48797	GO:0030029	GO Biological Processes	actin filament-based process	113	5.85	-12.95	-8.73
			GO:0050953	GO Biological Processes	sensory perception of light stimulus	46	2.38	-11.15	-7.41
			GO:009866	GO Biological Processes	inorganic cation transmembrane	104	5.39	-9.71	-6.26

2		transport				
GO:0006816	GO Biological Processes	calcium ion transport	65	3.37	-9.14	-5.79
GO:1905114	GO Biological Processes	cell surface receptor signaling pathway involved in cell-cell signaling	77	3.99	-8.88	-5.62
GO:0040008	GO Biological Processes	regulation of growth	88	4.56	-8.78	-5.56
GO:0034330	GO Biological Processes	cell junction organization	91	4.72	-8.70	-5.56
GO:0007423	GO Biological Processes	sensory organ development	77	3.99	-8.56	-5.49
GO:0006820	GO Biological Processes	anion transport	70	3.63	-7.68	-4.74
GO:0007264	GO Biological Processes	small GTPase mediated signal transduction	69	3.58	-7.54	-4.64
hsa04810	KEGG Pathway	Regulation of actin cytoskeleton	38	1.97	-7.35	-4.53
GO:0060627	GO Biological Processes	regulation of vesicle-mediated transport	71	3.68	-7.32	-4.53
GO:0046486	GO Biological Processes	glycerolipid metabolic process	57	2.95	-7.21	-4.47
GO:0048729	GO Biological Processes	tissue morphogenesis	78	4.04	-6.87	-4.17
GO:0007605	GO Biological Processes	sensory perception of sound	30	1.55	-6.75	-4.08
GO:0009792	GO Biological Processes	embryo development ending in birth	79	4.09	-6.75	-4.08

					or egg hatching				
			GO:0006936	GO Biological Processes	muscle contraction	51	2.64	-6.67	-4.02
			GO:0009636	GO Biological Processes	response to toxic substance	42	2.18	-6.63	-4.01
			hsa04510	KEGG Pathway	Focal adhesion	35	1.81	-6.62	-4.01
			GO:0006814	GO Biological Processes	sodium ion transport	40	2.07	-6.56	-3.97
3rd Components	1512	23120	GO:0050907	GO Biological Processes	detection of chemical stimulus involved in sensory perception	88	5.84	-23.62	-19.70
			GO:0042110	GO Biological Processes	T cell activation	75	4.98	-16.01	-12.79
			GO:0050778	GO Biological Processes	positive regulation of immune response	83	5.51	-15.74	-12.56
			hsa04660	KEGG Pathway	T cell receptor signaling pathway	30	1.99	-13.99	-10.92
			GO:0009617	GO Biological Processes	response to bacterium	88	5.84	-11.47	-8.57
			hsa04060	KEGG Pathway	Cytokine-cytokine receptor interaction	45	2.99	-10.95	-8.16
			GO:0045058	GO Biological Processes	T cell selection	18	1.19	-10.39	-7.69
			GO:0019079	GO Biological Processes	viral genome replication	28	1.86	-9.58	-7.00

GO:0002683	GO Biological Processes	negative regulation of immune system process	57	3.78	-9.44	-6.89
GO:0034612	GO Biological Processes	response to tumor necrosis factor	40	2.65	-9.11	-6.60
hsa04650	KEGG Pathway	Natural killer cell mediated cytotoxicity	27	1.79	-8.74	-6.29
GO:0031347	GO Biological Processes	regulation of defense response	69	4.58	-7.97	-5.59
GO:0002863	GO Biological Processes	positive regulation of inflammatory response to antigenic stimulus	8	0.53	-7.57	-5.26
GO:0002181	GO Biological Processes	cytoplasmic translation	26	1.73	-7.09	-4.87
hsa05169	KEGG Pathway	Epstein-Barr virus infection	40	2.65	-7.02	-4.82
GO:0055065	GO Biological Processes	metal ion homeostasis	68	4.51	-6.73	-4.56
GO:0009615	GO Biological Processes	response to virus	45	2.99	-6.62	-4.46
GO:0031424	GO Biological Processes	keratinization	15	1.00	-6.58	-4.43
GO:0006412	GO Biological Processes	translation	73	4.84	-6.51	-4.37
GO:0072538	GO Biological Processes	T-helper 17 type immune response	12	0.80	-6.41	-4.28

6th components	560	8617	GO:0009593	GO Biological Processes	detection of chemical stimulus	45	8.09	-16.04	-11.82
			GO:0009617	GO Biological Processes	response to bacterium	41	7.37	-8.29	-5.07
			GO:0001906	GO Biological Processes	cell killing	18	3.24	-7.24	-4.10
			GO:0070374	GO Biological Processes	positive regulation of ERK1 and ERK2 cascade	18	3.24	-6.32	-3.22
			GO:0009611	GO Biological Processes	response to wounding	30	5.40	-6.14	-3.07
			GO:0045862	GO Biological Processes	positive regulation of proteolysis	22	3.96	-5.16	-2.26
			GO:0061041	GO Biological Processes	regulation of wound healing	12	2.16	-4.83	-2.06
			GO:0022409	GO Biological Processes	positive regulation of cell-cell adhesion	18	3.24	-4.79	-2.04
			GO:0031623	GO Biological Processes	receptor internalization	11	1.98	-4.78	-2.04
			GO:0046942	GO Biological Processes	carboxylic acid transport	17	3.06	-4.44	-1.83
			GO:0050778	GO Biological Processes	positive regulation of immune response	27	4.86	-4.44	-1.83
			GO:0098742	GO Biological Processes	cell-cell adhesion via plasma-membra	17	3.06	-4.42	-1.83

				ne adhesion molecules				
		GO:0002274	GO Biological Processes	myeloid leukocyte activation	15	2.70	-4.34	-1.80
		GO:0052548	GO Biological Processes	regulation of endopeptidase activity	22	3.96	-4.20	-1.72
		GO:0044403	GO Biological Processes	biological process involved in symbiotic interaction	17	3.06	-4.06	-1.62
		GO:0010811	GO Biological Processes	positive regulation of cell-substrate adhesion	10	1.80	-3.77	-1.46
		GO:0042554	GO Biological Processes	superoxide anion generation	6	1.08	-3.67	-1.39
		GO:0008202	GO Biological Processes	steroid metabolic process	17	3.06	-3.64	-1.37
		GO:0051092	GO Biological Processes	positive regulation of NF-kappaB transcription factor activity	11	1.98	-3.62	-1.35
		GO:1904141	GO Biological Processes	positive regulation of microglial cell migration	3	0.54	-3.60	-1.35
13th components	37	GO:0000377	GO Biological Processes	RNA splicing, via transesterification reactions with bulged	4	10.81	-3.10	0.00

			adenosine as nucleophile				
	GO:2000377	GO Biological Processes	regulation of reactive oxygen species metabolic process	3	8.11	-2.94	0.00
	GO:0006091	GO Biological Processes	generation of precursor metabolites and energy	4	10.81	-2.42	0.00

Abbreviation: KEGG, Kyoto Encyclopedia of Genes and Genomes. GO, gene ontology.
